# Evaluation of factors influencing tick bites and tick-borne infections: a longitudinal study

**DOI:** 10.1186/s13071-021-04751-0

**Published:** 2021-05-29

**Authors:** Bo Bødker Jensen, Mie Topholm Bruun, Per Moestrup Jensen, Andreas Kristian Pedersen, Pierre-Edouard Fournier, Sigurdur Skarphedinsson, Ming Chen

**Affiliations:** 1 Department of Clinical Microbiology, Hospital of Southern Jutland, Sydvang 1, 6400 Sønderborg, Denmark; 2 Clinical Centre for Emerging and Vector-Borne Infections, Odense University Hospital, Odense, Denmark; 3 Faculty of Health Sciences, University of Southern Denmark, Odense, Denmark; 4 Department of Clinical Immunology, Odense University Hospital, Odense, Denmark; 5 Faculty of Science, University of Copenhagen, Copenhagen, Denmark; 6 Research and Education Center, Hospital of Southern Jutland, Aabenraa, Denmark; 7 Aix-Marseille University, IRD, AP-HM, SSA, VITROME, Marseille, France; 8 IHU-Mediterranée Infection, Marseille, France; 9 Department of Infectious Diseases, Odense University Hospital, Odense, Denmark

**Keywords:** Tick-borne infections, Tick, Borreliosis, Rickettsiosis, Climate, Symptoms, Seroconversion, Prevalence

## Abstract

**Background:**

Various tick-borne infections like borreliosis and rickettsiosis pose a health risk to humans in many parts of the world. We investigated seroprevalence of and seroconversion to *Borrelia burgdorferi* and *Rickettsia* spp. and relation to tick-bites, weather and clinical manifestations in Denmark.

**Methods:**

Blood donors were enrolled at the Hospital of Southern Jutland in June–July with follow-up November–February of 2018 and 2019. Blood samples were collected, and a questionnaire regarding tick bites, potential exposures and symptoms was completed at each visit. Samples were tested for presence of IgM and IgG antibodies directed against *B. burgdorferi* and *Rickettsia* spp. using *R. helvetica* and *R. felis* as antigens. Data were examined for correlation between tick bites, serological results, potential exposures and symptoms.

**Results:**

Two-hundred and fourteen (93 follow-ups) and 130 (38 follow-ups) blood donors were included in 2018 and 2019, respectively. The total borrelia seroconversion rate was 6.3% (CI 2.1–10.5), while the prevalence of IgM and IgG antibodies was 7.8% (CI 4.9–10.6) and 6.7% (CI 4–9.3), respectively. Seroconversion to *Rickettsia* spp. was detected in one participant. Tick bites and seroconversion were not significantly associated with the reported unspecific symptoms, but unspecific symptoms were common in the study population. There was no significant difference in number of tick bites or seroconversion/prevalence between seasons with highly alternating weather.

**Conclusions:**

Results suggest that weather conditions in an individual year have a limited impact. Anti-*Borrelia*-antibodies do not seem to persist in serum for several years. Rickettsiosis is of limited concern in Denmark.

**Graphical Abstract:**

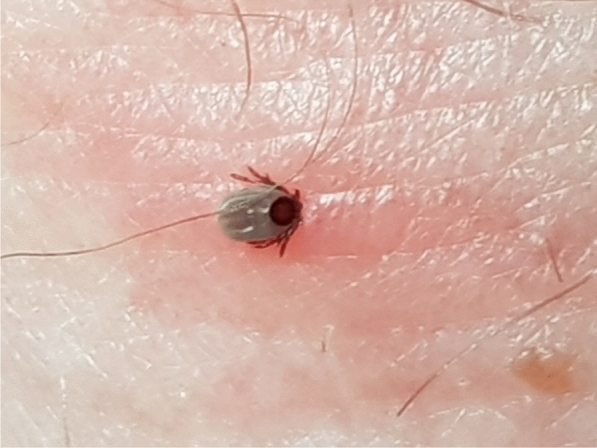

**Supplementary Information:**

The online version contains supplementary material available at 10.1186/s13071-021-04751-0.

## Background

The transmission of various infectious agents by ticks poses a risk to human health in many parts of the world. The most well-known is probably *Borrelia burgdorferi* s.l., which is among the most common causes of tick-borne infection in Europe and North America, but the actual incidence is uncertain due to heterogenic surveillance systems without mandatory notification [1]. However, a number of other tick-borne pathogens cause disease in humans and are dominant causes in other parts of the world, as well as being present in Europe and North America. These include among others rickettsiosis, babesiosis, human anaplasmosis and tick-borne encephalitis. Tick-borne infections may present with unspecific symptoms including fever, headache, myalgia, arthralgia and fatigue [2–4], in addition to more specific symptoms, like an eschar or a typical rash in rickettsiosis [3] and an erythema migrans in borreliosis [2]. The tick *Ixodes ricinus* is present in many parts of Europe [5], and it is the primary tick species of human concern in Denmark. *I. ricinus* serves as a vector for various infectious agents and is known to feed readily on a wide variety of hosts, including humans [6]. The tick requires a humid microclimate during its life cycle and to remain active while seeking a blood host [7]. Generally, it is accepted that the incidence of tick-borne infections is influenced by changing weather conditions [8]. Two of the most abundant microorganisms found in Danish ticks are *B. burgdorferi* and *Rickettsia helvetica* [9–16], which cause infections in humans of varying severity.

Borreliosis may progress through stages, usually with initial multiplication and local migration of the spirochete at the bite site causing inflammation with erythema migrans (EM) and sometimes fever. It can be followed by infection of the nervous system causing subacute meningitis with symptoms including headache, radicular pain, peripheral paresis, fatigue a.m. [17–19], infections of the joints causing arthritis [20], or infection of the heart causing borrelia carditis with potential atrioventricular blockage [21] and late-stage infection presenting as acrodermatitis chronica atrophicans or progressed nervous system infection. The annual incidence of neuroborreliosis in Denmark is approximately 3/100,000 [22].

Infection with *R. helvetica* is usually asymptomatic or with a mild febrile illness and/or flu-like symptoms of headache, myalgia and arthralgia, and potentially with an inoculation eschar or a rash [9, 23–26], but rare cases of subacute meningitis and myocarditis have been reported [27–29].

Diagnosis of borreliosis and rickettsiosis usually relies on serological analysis, preferably with an additional test of convalescent serum though this is rarely obtained [30]. However, erythema migrans (EM) is usually a clinical diagnosis with a limited value of serological testing [31], and for proper diagnosis of neuroborreliosis testing of the cerebrospinal fluid is recommended [32]. For rickettsiosis, a biopsy/swab from the eschar, if present, may be analysed using polymerase chain reaction [33, 34], confirming the infection before the development of an antibody response.

Serological studies in various populations and studies of tick-bitten individuals have shown a high, but variable anti-*B. burgdorferi* antibody prevalence indicating that transmission rarely progresses to more severe infections [9, 26, 35–39]. Evaluation of the significance of these findings may be more accurately assessed with studies of seroconversion rates, symptoms and risk factors in the general population. Unfortunately, studies of this type are scarce.

The objective of this study was to estimate the seroprevalence and seroconversion rates for borreliosis and rickettsiosis and to evaluate their relationship with the symptoms in Southern Jutland, Denmark.

The arid Danish summer of 2018 and rainy summer of 2019 provided an ideal opportunity to investigate whether the contrasting weather conditions influenced human exposure to ticks and tick-borne infections.

## Methods

### Study population

Two groups of blood donors from the blood bank at the Hospital of Southern Jutland, Sønderborg were enrolled prospectively during two consecutive tick seasons. Enrollment of the first group occurred during June–July of 2018 with follow-up in November–February. The second group was enrolled during June–July of 2019 with follow-up in November–February. Blood donors are requested to donate only when there is a need for their respective blood type in the blood bank. Due to this, the follow-up was limited by finite necessity for blood components, since blood sample collection was only done at visits to the blood bank.

At each visit, the participants provided a blood sample of approximately 4 mL. The samples were centrifuged at 3000 rpm for 10 min, and the serum was stored in aliquots at −20 °C until analysis to avoid multiple freeze-thaw cycles.

### Questionnaire

At inclusion, participants gave informed consent and filled out a questionnaire regarding the following:

Background (age, sex), exposures (tick bites in the preceding year or since the first blood sample and date of these, outdoor activities, pet ownership), symptoms potentially associated with a tick-borne infection or of unknown origin in the preceding 3 months or since the first blood sample (fever, headache, myalgia, arthralgia, lymphadenitis, dermatological changes, other symptoms). The study setting was based on participants answering the questionnaire directly before donating blood, so the questionnaire had to be short and straightforward. The participants did not have time to answer a lengthy questionnaire without prior planning. Therefore, it was not possible to include detailed answers to questions of time spent outdoors, exact locations (are there risks of tick bites in the area), etc.

### Serological analyses

Serum was tested for anti-*B. burgdorferi* IgM and IgG antibodies on the Liaison XL system using a chemiluminescence immunoassay (DiaSorin, Saluggia, Italy) according to the manufacturer’s instructions. The IgM analysis uses *B. burgdorferi* VIsE and OspC as the antigen, and the IgG uses VIsE. An IgM antibody concentration > 22 AU/mL and IgG > 15 AU/mL were considered positive (AU = arbitrary units of light measured in the chemiluminescence reaction).

Anti-rickettsial antibodies were tested with immunofluorescence microscopy using a Zeiss Axioscope with fluorescence filter connected with an HXP 120 C lighting unit (Carl Zeiss AG, Oberkochen, Germany). For the analysis of IgM the serum was first treated with a rheumatoid factor absorbent and an IgG stripper (Bio-Rad Laboratories, Hercules, USA). The 12 well slides (Erie Scientific LCC, Portsmouth, USA) were prepared with *R. helvetica* and *R. felis* antigen and fixated with 99% methanol. These slides were then successively incubated with the human serum and fluorescein isothiocyanate coupled anti-human IgM and IgG goat serum (Bio-Rad) diluted 1:200 in a phosphate-buffered saline (Sigma-Aldrich, Saint Louis, USA), skimmed milk (Sigma-Aldrich) and Evans blue 1% (Bio-Rad) solution. Slides were mounted using Fluoromount aqueous mounting medium (Sigma-Aldrich) before microscopy. The serum was screened at titers 1:16 and 1:32 with further twofold serial dilution if positive at these titers. A titer ≥ 1:64 was considered positive for IgG and ≥ 1:32 for IgM. Antigens and positive control serum for spotted fever group rickettsiae (titers 1:64 IgG and 1:16 IgM) were provided by the French Reference Center for Rickettsioses (FRCR, Méditerranée Infection, Marseille, France), negative control serum was from a healthy donor confirmed negative for anti-rickettsial antibodies at the FRCR. *R. helvetica* was chosen as an antigen since it is the only rickettsial species proven firmly established in Denmark. *R. felis* was chosen to evaluate potential serological evidence of this species presence in Denmark.

### Statistical analyses

The demographic variables were summarized using descriptive statistics. *χ*^2^-test or Fischer’s exact was used for the categorical variables depending on Cochran's rule and Wilcoxon rank-sum test or *t*-test for non-categorical variables were used depending on the distribution of the variable. The difference between the prevalence of anti-*Borrelia burgdorferi* IgG and seroconversion was investigated using a −2 log *Q* test. To analyze the prevalence of tick bites between seasons, logistic regression was performed fulfilling the one in ten rule to avoid overfitting. The correlation structure between the variables anti-*Borrelia burgdorferi* antibodies (IgM, IgG), reported tick bite, outdoor recreational activities, outdoor work-related activities, pet (dog, cat), age and sex were explored using exploratory polychoric factor analysis. The number of underlying factors in the factor analysis were identified through Horn’s parallel analysis. All statistical analyses were done with Stata version 16, and a *p*-value < 0.05 was considered statistically significant. No correction for multiple testing was utilized.

## Results

### Baseline data

Three hundred forty-four blood donors were enrolled during June–July of 2018–19 with follow-up of 131 in the post-season period November–February. In 2018, 214 participants were included with 93 available for follow-up, while 130 were included in 2019, with 38 available for follow-up. Four follow-up samples in the 2019 season were unfit for testing due to improper storage of the samples. The median age of the participants was 46 years, and 43.9% were female. More than half of the participants had one or more risk factors of tick exposure (Table 1).

**Table 1:** Baseline and serology data

Baseline	2018	2019	*P*-value	Total
Age, years, median (range)	45 (19–65)	46.5 (21–67)	0.76	46 (19–67)
Sex, male/female	112/102	81/49	0.071	193/151
Outdoor recreational activities, no. (%)	150/214 (70.1%)	96/130 (73.8%)	0.45	246 (71.3%, 95% CI 66.5–76.1)
Outdoor work-related activities, no. (%)	54/214 (25.2%)	31/130 (23.8%)	0.708	85 (24.7%, 95% CI 20.1–29.3)
Furred pet ownership, no. (%)	108/214 (50.47%)	57/130 (43.85%)	0.474	165 (47.9%, 95% CI 42.6–53.1)
Tick bite within season, no. (%)	15/214 (7.01%)	7/130 (5.38%)	0.55	22 (6.4%, 95% CI 3.8–9)
*Borrelia burgdorferi*				
IgM sample 1, no. (%)	14/214 (7.48%)	13/130 (10%)	0.293	27 (7.84%, 95% CI 4.9–10.6)
IgG sample 1, no. (%)	10/214 (4.67%)	13/130 (10%)	0.069	23 (6.7%, 95% CI 4–9.3)
Seroconversion, no. (%)^a^	6/93 (6.45%)	2/34 (5.88%)	1	8/127 (6.3%, 95% CI 2.1–10.5)
*Rickettsia* spp. (antigen:* R. helvetica*)				
IgM sample 1, no. (%)	0	0		0
IgG sample 1, no. (%)	0	0		0
Seroconversion, no. (%)	0	1/34 (2.9%)		1/127 (0.8%, 95% CI 0.02–4.3)^b^
Anti-*Rickettsia felis* antibodies	0	0		0

^a^Seroconversion was defined as detection of antibodies (IgM and/or IgG) in the follow-up sample not found in the primary sample, a change from IgM to IgG antibodies, or doubling of the AU in the second sample

^b^Clopper-Pearson was used for this analysis since Cochran's rule was not fulfilled

*AU* arbitrary units of light

### Serology

The prevalence of anti-*B. burgdorferi* IgG antibodies (6.7%) was not significantly different from the number of participants that seroconverted (6.3%) with a −2 log Q test providing a *p*-value of 0.39.

Of the eight participants seroconverting to *B. burgdorferi* (Table 1), seven developed IgM antibodies between the two samples (participants no. 6, 48, 50, 51, 52, 53, 54), and one participant developed IgG antibodies (participant no. 49). Three of the participants who were positive for IgM at the first blood test additionally tested positive for IgG (participants no. 1, 3, 19) (Table 2); one of these participants reported a tick bite within the last year, and another reported 3 days of febrile illness without other symptoms within the last 3 months. No follow-up samples were available for these participants.

**Table 2:** AU-values of anti-*B. burgdorferi* antibody-positive blood samples

	Sample 1		Sample 2	
Participant	IgM	IgG	IgM	IgG
1	47.04	15.92	NF^a^	NF
2	Neg^a^	24.73	Neg	27.05
3	22.37	17.95	NF	NF
4	Neg	15.99	Neg	15.58
5	Neg	200.5	NF	NF
6	Neg	41.33	22.47	46.05
7	Neg	96.41	NF	NF
8	Neg	> 240	NF	NF
9	Neg	20.27	NF	NF
10	Neg	18.73	NF	NF
11	Neg	39.13	NF	NF
12	Neg	100.0	NF	NF
13	Neg	56.56	NF	NF
14	Neg	58.45	NF	NF
15	Neg	35.57	NF	NF
16	Neg	19.88	Neg	Neg
17	Neg	18.99	NF	NF
18	Neg	19.71	NF	NF
19	53.7	42.21	NF	NF
20	Neg	> 240	Neg	185.6
21	Neg	63.5	NF	NF
22	Neg	97.0	NF	NF
23	Neg	15.61	NF	NF
24	43.3	Neg	34.73	Neg
25	29.94	Neg	NF	NF
26	34.8	Neg	NF	NF
27	23.59	Neg	Neg	Neg
28	26.55	Neg	NF	NF
29	38.86	Neg	41.43	Neg
30	33.77	Neg	31.69	Neg
31	37.51	Neg	46.91	Neg
32	56.7	Neg	NF	NF
33	35.25	Neg	NF	NF
34	30.75	Neg	24.06	Neg
35	39.87	Neg	28.24	Neg
36	26.93	Neg	NF	NF
37	43.69	Neg	NF	NF
38	50.64	Neg	62.61	Neg
39	27.91	Neg	28.18	Neg
40	107.4	Neg	NF	NF
41	35.01	Neg	32.63	Neg
42	95.39	Neg	NF	NF
43	22.8	Neg	Neg	Neg
44	24.17	Neg	23.34	Neg
45	22.53	Neg	NF	NF
46	23.44	Neg	23.74	Neg
47	44.04	Neg	NF	NF
48	Neg	Neg	104.0	Neg
49	Neg	Neg	Neg	43.4
50	Neg	Neg	25.67	Neg
51	Neg	Neg	26.95	Neg
52	Neg	Neg	83.55	Neg
53	Neg	Neg	36.27	Neg
54	Neg	Neg	32.76	Neg

^*a*^*NF* no follow-up blood sample available, *Neg* negative test

Two participants who tested positive for IgM in the primary sample were negative in the follow-up sample and did not develop IgG antibodies. Eleven participants had positive IgM in both samples, ranging from 23.34 to 62.61 AU, without developing IgG and with a maximum AU difference of ± 12 between the first and second sample (participants no. 24, 29, 30, 31, 34, 35, 38, 39, 41, 44, 46) (Table 2).

The only anti-*Rickettsia* spp. antibody-positive participant (IgM titer *R. helvetica*: 1:128, *R. felis*: 1:16) was a 55-year-old woman without symptoms who seroconverted in 2019. She had furred and feathered household animals (cats, hen and ducks), and practiced outdoor recreational activities. She had a positive anti-*B. burgdorferi* IgM antibody test in her blood samples without titer rise or positive IgG in the follow-up sample, and suffered from rheumatoid arthritis.

No participants had anti-*R. felis* antibodies at an above cut-off level.

### Association of symptoms and risk factors with tick bites and seroconversion

As expected, there was a significant difference in the reported wounds of unknown cause or after insect bite between tick-bitten and non-tick-bitten participants, but there was no significant difference in the other reported symptoms (Table 3). We found no significant difference in reported symptoms between participants who seroconverted and those who did not. There was no significant relationship between tick bites or seroconversion among participants with potential risk factors (Table 3).

**Table 3:** Association of symptoms and potential risk factors with tick bites and seroconversion

	No tick bite (no. = 322)/tick bite (no. = 22)^a^, *P*-value	Total
Symptoms ≤ 3 month		
Fever	4/0, *P* = 1	4/344 = 1.2%
Headache	59/3, *P* = 0.777	62/344 = 18%
Myalgia	19/2, *P* = 0.634	21/344 = 6.1%
Arthralgia	18/1, *P* = 1	19/344 = 5.5%
Lymphadenitis	10/1, *P* = 0.522	11/344 = 3.2%
Fatigue	1/0, *P* = 1	1/344 = 0.3%
Wound, unknown cause or after insect bite	21/5, *P* = 0.04	26/344 = 7.6%
Wound, black crust	4/1, *P* = 0.411	5/344 = 1.5%
Red ring on the skin	7/1, *P* = 0.520	8/344 = 2.3%
Rash	7/1, *P* = 0.520	8/344 = 2.3%
Potential risk factors		
Outdoor recreational activities	228/18, *P* = 0.335	246/344 = 71.5%
Outdoor work related activities	76/9, *P* = 0.077	85/344 = 24.7%
Furred pet ownership	152/14, *P* = 0.136	166/344 = 48.3%

	No seroconversion (no. = 119)/seroconversion (no. = 8), *P*-value	
Symptoms since last blood sample		
Fever	1/0, *P* = 1	1/127 = 0.8%
Headache	8/0, *P* = 1	8/127 = 6.3%
Myalgia	3/0, *P* = 1	3/127 = 2.4%
Arthralgia	5/0, *P* = 1	5/127 = 3.9%
Lymphadenitis	0/0	0/127
Fatigue	6/0, *P* = 1	6/127 = 4.7%
Wound, unknown cause or after insect bite	3/0, *P* = 1	3/127 = 2.4%
Wound, black crust	0/0	0/127
Red ring on the skin	0/0	0/127
Rash	0/0	0/127
Potential risk factors		
Outdoor recreational activities	81/4, *P* = 0.267	85/127 = 66.9%
Outdoor work related activities	30/1, *P* = 0.678	31/127 = 24.4%
Furred pet ownership	59/3, *P* = 0.717	62/127 = 48.8%
Tick bite within season	7/0, *P* = 1	7/127 = 5.5%

^a^Tick bite in the period before the first sample collection and in the same season

### Polychoric factor analysis

The factor analysis identified a correlation between reported tick bite(s) and anti-*B. burgdorferi* IgG. A correlation between dog ownership and outdoor recreational activities was identified. A correlation between age, sex and outdoor work activities was also identified. A reverse correlation between cat ownership and anti-*B. burgdorferi* IgM was found. The factor analysis revealed no correlation between tick bites and potential risk factors, anti-*B. burgdorferi* IgM, or sex and age (Additional file 1).

### Comparison of seasons

There was no significant difference between the 2018 and 2019 tick seasons regarding anti-*B. burgdorferi* IgM- or IgG-positive participants, reported tick bites or seroconversion (Table 1). Logistic regression analysis of the difference in reported tick bites between 2018 and 2019 resulted in an odds ratio of 0.75 (95% CI = 0.30–1.90, *P* = 0.55).

As participants were asked about tick bites and dates of these 1 year before the first sample, this also gave some information about exposure from early June in the previous tick season. In the 2018 group, 15 participants reported tick bites in the current season and 14 in the 2017 tick season. In 2019, seven participants reported tick bites in the current season and eight in the 2018 season. The first reported bite in 2018 was on the 4th of May and in 2019 the 14th of March. Some answers about the dates were unspecific and mentioned only the year, season, month or time span, e.g. May/June.

## Discussion

In this study we investigated seroprevalence, seroconversion, tick bites, exposures and symptoms potentially related to tick-borne infection among Danish adults. Additionally, we investigated the impact of weather on the frequency of tick bites and tick-borne infections. We found no significant difference between anti-*B. burgdorferi* antibody seroprevalence and seroconversion and a correlation between reported tick bite and anti-*B. burgdorferi* IgG, but not IgM. Rickettsiosis was very rare. There was only very limited evidence of symptomatic tick-borne infection in the investigated population, but frequent unspecific symptoms. There was no difference in reported tick bites or tick-borne infections between the investigated seasons, despite highly variable weather.

The prevalence of anti-*B. burgdorferi* IgG was not significantly higher than the incidence shown by the seroconversion rate. This indicates that anti-*B. burgdorferi* antibodies do not remain in the blood to a level above the cut-off of serological analysis for several years unless reactivated by a new infection. If antibodies persisted for 2 or more years, the prevalence would be at least double the incidence. This finding accounts for serological analysis and cannot be transferred to the intrathecal antibody test, as this is relying on the index of antibody amount between serum and cerebrospinal fluid. This may be positive when the serological analysis is negative [40] and it has been proposed to remain positive for years [41]. The IgG seroprevalence in our study is in concordance with previous blood donor data from the Region of Southern Denmark [42]. Eleven IgM-positive participants were continuously positive in the follow-up without significant titer change or development of IgG antibodies, and the factor analysis found no correlation between tick bites and the presence of anti-*B. burgdorferi* IgM antibodies in serum. This could indicate that cautions should be taken regarding clinical interpretation of serum IgM antibodies as previously suggested [35], especially without relevant symptoms or a follow-up blood sample.

There was only one participant positive for anti-*Rickettsia* spp. antibodies, and she was asymptomatic, which indicates that autochthonous rickettsiosis is currently of least concern to the public health in the region. In Denmark *B. burgdorferi* and *R. helvetica* are common, indicating that the transmission of, or infectious/pathogenic potential for *B. burgdorferi* is higher than for *R. helvetica*, as one study that included Southern Jutland reported nearly equal prevalence of the two bacteria in sampled ticks [13]. The result is in concordance with a study showing that most recorded cases of rickettsiosis in Denmark are due to imported African tick bite fever usually caused by *R. africae* [30]. The investigated region of Southern Jutland is particularly interesting regarding tick-borne infections since this is the primary region exposed to migrating land-based animals from the European mainland bringing new tick species and pathogens with them [43]. As *R. helvetica* is the only rickettsial species being reported in nearly all studies on Danish ticks [9–12, 14–16], with single findings of *Rickettsia massiliae* [13] and *Rickettsia raoultii* [43] being the exceptions, *R. helvetica* is the likely cause of the seroconversion. However, due to antibody cross-reactivity between rickettsial species, it is not absolutely certain. There was no serological evidence of *R. felis* being present in the studied area of Denmark, though its presence has been reported in neighboring Germany [44–46] and Sweden [47, 48].

We found no significant difference between reported symptoms, except for wound after an insect bite or of unknown causes, in groups exposed to tick bites or with seroconversion indicating that in some instances symptoms, which may be related to tick-borne infections are common in the general population, e.g. headache, myalgia and arthralgia, and hence unspecific. It also indicates that symptoms more related to infection, like fever and EM, are rare even after exposure to ticks or the tick-borne pathogens present in our region of Denmark. These findings correlate with results from Holland, Switzerland and Sweden [26, 37, 49]. However, the design does impose a risk of recall bias and under-reporting, as mild symptoms may not be remembered or noted (e.g. low fever) and the bitten person does not necessarily acknowledge a tick bite shown by the number of neuroborreliosis patients reporting tick bites [18, 19].

Three participants were both anti-*B. burgdorferi* IgM- and IgG-positive at the primary blood sample, which indicates recent exposure, with only one of these reporting a tick bite and another reporting having experienced 3 days of self-limiting fever and no other symptoms within the last 3 months. The participant with short-term fever could be the only participant with potential symptomatic tick-borne infection. Unfortunately, no follow-up blood samples were obtained from these individuals.

The simplified questionnaire without registration of amount of time spent outdoors and exact location explains the deviance from the fact that people who spend a lot of time in tick-infested areas are expectedly at an increased risk of being bitten, even though spending time outdoors, in general, did not seem to be a risk factor. Furred animals could potentially pose a risk of tick bites in some settings as they could act as hosts for ticks and carry these to the proximity of humans. However, the animals need to be exposed to ticks to do this. Unfortunately, the study design made it impossible to distinguish between animals exposed to ticks and not, though animals noted as strictly indoor pets were excluded from analysis along with lizards. Many unrecorded factors can influence human exposure to ticks, but owning a furred animal does not seem to be an isolated risk factor. A Scandinavian study on risk factors neither found an association between pet ownership and reported tick bites, but between outdoor activities and reported tick bites [50].

The summer of 2018 was among the most arid in recent Danish history. It was preceded by a winter and spring with snow until April. In contrast, the summers of 2017 and 2019 had considerably more rainfall and higher air humidity [51]. These weather conditions would be expected to increase the number of tick bites and hence tick-borne infections in 2019. However, there were no significant differences between the two seasons in either tick bites or seroconversion among the participants. In addition, there were no differences in the number of reported tick bites in each group from 2018 to 2019. Within-season weather fluctuations have previously been suggested as a factor, influencing the amount of tick-human interaction [52] though, in our study, the overall difference between full seasons was investigated. The lack of difference coincides with data suggesting relatively stable seasonal distribution of reported neuroborreliosis cases over more extended periods [53], with the primary influence being access to roe deer, the main feeding and mating host [54].

It is important to note that the results are not an indicator of tick activity in nature. They are an indicator of the interface between humans and ticks. This is influenced by differences in human behavior under different weather conditions, including time spent in nature and, perhaps more importantly, the behavior when time is spent in nature. The observations are based on a geographical setting where *I. ricinus* is the only evidently established tick species of human concern. This must be kept in mind if the weather impact results are applied to settings with presence of other tick species.

## Conclusions

Some precautions must be taken when interpreting the findings in this study, due to sampling size and study design, and subsequent risk of reporting and recall bias. We found that anti-*B. burgdorferi* antibodies do not seem to remain measurable to an above cut-off level for several years, which is essential when evaluating serological analysis in a clinical setting and prompts further studies on the subject.

The presence of solitary anti-*B. burgdorferi* IgM antibodies in serum, without relevant symptoms or a recent prior/follow-up sample for comparison, should be interpreted with caution.

We found no serological evidence of *R. felis*, and rickettsiosis is of least concern in Southern Jutland, Denmark, but continued surveillance is recommended.

Reported symptoms due to tick bites or tick-borne pathogens are relatively rare in the general adult population in the region, but unspecific symptoms are common, which must be kept in mind when evaluating potential patients.

Additionally, short-term weather fluctuations from year to year seem to have limited impact on the prevalence of tick bites and tick-borne infections in a Danish setting due to other contributing factors, but further studies are needed to elucidate this, and the long-term picture remains uncertain.

## Supplementary Information

**Additional file 1: Table S1.** Results from the polychoric factor analysis of correlated variables in the five underlying factors, which were identified through Horn’s parallel analysis. The factors are to be seen as underlying variables, which investigate the same aspects as our measurable variables. Values with an absolute value over 0.20 indicate that the measured variable is associated with the underlying factor.

## Data Availability

The datasets used and analysed during the current study are available from the corresponding author on reasonable request in an anonymized form.
