# Supplementary material for: Evaluation of factors influencing tick bites and tick-borne infections: a longitudinal study
Source: Parasit Vectors. 2021 May 29;14:289. doi: 10.1186/s13071-021-04751-0 (PMC8164064; doi:10.1186/s13071-021-04751-0)
Supplement: Supplementary file 1 — Additional file 1: Table S1. Results from the polychoric factor analysis of correlated variables in the five underlying factors, which were identified through Horn’s parallel analysis. The factors are to be seen as underlying variables, which investigate the same aspects as our measurable variables. Values with an absolute value over 0.20 indicate that the measured variable is associated with the underlying factor. [file 13071_2021_4751_MOESM1_ESM.docx]

**Evaluation of Factors Influencing Tick bites and Tick-borne Infections: A Longitudinal Study**

Appendix:

**Table S1.** Results from the polychoric factor analysis of correlated variables in the five underlying factors which were identified through Horn’s parallel analysis. The factors are to be seen as underlying variables, which investigate the same aspects as our measurable variables. Values with an absolute value over 0.20 indicate that the measured variable is associated with the underlying factor.

| Variabel | Factor1 | Factor2 | Factor3 | Factor4 | Factor5 |
| --- | --- | --- | --- | --- | --- |
| Anti-Borrelia burgdorferi IgM | 0.195 | 0.003 | **-0.515** | 0.166 | -0.038 |
| Anti-Borrelia burgdorferi IgG | -0.122 | -0.096 | -0.053 | **0.581** | 0.006 |
| Reported tick bite | 0.174 | 0.023 | 0.110 | **0.217** | 0.120 |
| Recreational outdoor activities | **0.594** | 0,022 | -0.053 | -0.114 | 0.026 |
| Outdoor during work hours | 0.073 | **0.229** | -0.015 | 0.170 | **0.205** |
| Pet (dog) | **0.290** | -0.143 | 0.018 | -0.178 | 0.198 |
| Pet (cat) | 0.085 | -0.026 | **0.523** | 0.030 | -0.031 |
| Age | 0.167 | **0.360** | 0.125 | 0.068 | -0.117 |
| Sex (male) | -0.072 | **0.516** | -0.138 | -0.154 | 0.042 |
